# Polycystic ovaries and herbal remedies: A systematic review

**DOI:** 10.5935/1518-0557.20220024

**Published:** 2023

**Authors:** Aliasghar Manouchehri, Saber Abbaszadeh, Mahdeyeh Ahmadi, Fateme Khajoei Nejad, Mahmoud Bahmani, Neda Dastyar

**Affiliations:** 1 Assistant Professor, Department of Internal Medicine, Shahid Beheshti Hospital, Babol University of Medical Sciences, Babol, Iran; 2 Department of Biochemistry And Genetics, School of Medicine, Lorestan University of Medical Sciences, Khorramabad, Iran; 3 Medical School, Jiroft University of Medical Sciences, Jiroft, Iran; 4 Nursing and Midwifery School, Kerman University of Medical Sciences, Kerman, Iran; 5 Biotechnology and Medicinal Plants Research Center, Ilam University of Medical Sciences, Ilam, Iran; 6 Department of Midwifery, Nursing and Midwifery School, Jiroft University of Medical Sciences, Jiroft, Iran

**Keywords:** polycystic ovary, medicinal plants, infertility, hormones

## Abstract

**Objective:**

Polycystic ovary syndrome (PCOS) is an endocrine disorder that affects one in every 15 women worldwide. This disorder is mainly characterized by increased levels of male hormones (androgens), acne, and hirsutism, and can lead to long-term insulin resistance, miscarriage, or even infertility in women. PCOS is a disorder that can be treated with natural and allopathic remedies that work against the PCOS mechanism. The present study reviews previous studies on the treatment of PCOS using natural drugs.

**Methods:**

The data in this study were collected from articles published in reputable databases including ScienceDirect, PubMed, Google Scholar, and SID in the field of medicinal plants from 1990 to 2021.

**Results:**

A review of the literature showed that plants such as aloe vera and chamomile improve fertility by increasing the number of ovarian follicles. Besides, Vitex agnus-castus and octane reduce hirsutism by reducing testosterone and androgen levels. It was also shown that liquorice, ginseng, cinnamon, and de chiro Inositol improve the adverse effects of diabetes caused by PCOS by lowering lipid and blood glucose levels. Moreover, Stachys lavandulifolia and fennel are effective in changing endometrial tissue parameters in PCOS by reducing estrogen and hyperplasia.

**Conclusions:**

Various studies have shown that herbal medicines can improve PCOS symptoms in women with minimal side effects but a longer treatment cycle.

## INTRODUCTION

The therapeutic effects and uses of herbs have been well established. A return to nature and the use of drugs of plant and natural origin takes place in a situation where modern man, by promoting the use of chemical drugs, has faced the side effects and complications of these drugs. Scientific studies have demonstrated the effectiveness and safety of several complementary medicine methods including herbs in the treatment of some diseases (Rashidi *et al.,* 2012).

Polycystic ovary syndrome (PCOS) is one of the most common endocrine disorders in women. It is a complex disease that, despite the rapid progress in research on it, has remained one of the most important challenges for researchers, physicians, health care providers, and health policymakers. Women with PCOS generally have large ovaries with hypertrophic stroma. These individuals do not ovulate despite having a large number of follicles due to impaired growth and atresia of the follicles. Clinical manifestations of this disease are varied and include menstrual disorders, clinical signs of androgen overload, obesity and infertility, hair loss, acne, insulin resistance resulting in type II diabetes, lipid disorder, systemic inflammation, oxidative stress, and long-term complications (Amini *et al*., 2015). The prevalence of this disease is reported to be 4 to 25%, depending on the definition provided. A national study reported its prevalence as 14.6% in Iran (Nasiri Amiri *et al*., 2013). To this end, the present study aimed to investigate the effect of natural compounds and medicinal plants effective in the treatment of polycystic ovaries.

## MEDICINAL PLANTS EFFECTIVE IN THE TREATMENT OF PCOS

### Aloe vera

Aloe vera, also known as *Aloe arborescens*, is a perennial herbaceous plant of the Liliaceae family. This plant contains vitamins A, C, and E. It also has antioxidant properties induced by reducing the level of lipid peroxidation. Aloe vera contains nutrients and minerals, salicylic acid, enzymes, tannins, and a variety of polysaccharides. Aloe vera gel contains mainly water and polysaccharides (pectins, cellulose, hemicellulose, glucomannan, skymann, and mannose derivatives). The active ingredients in the gel and leaves of this plant include aloe, asmodin, aloe asmodine, barbaloin, and polymonosaccharides such as sterols and organic acids (Akbar, 2020). Polysaccharide compounds in Aloe vera gel are able to reduce and repair inflammation. These compounds also have antibacterial and antimicrobial properties (Mezzetti *et al*., 2020). Aloe vera causes the number of primary germ cells in the ovary to become normal, and as a result, it can have beneficial and supportive effects on ovarian tissue and folliculogenesis (Yagi, 2015).

One study examined the effect of aloe vera gel on PCOS rats. Female rats were orally fed letrozole for 5 months and no nonsteroidal aromatase inhibitors were used to induce PCOS. The animals were then treated with 1 ml of aloe vera gel for 45 days. Stress cycle, glucose sensitivity, and steroidogenic activity were evaluated in the rats. The results showed that the use of a stimulant (letrozole) along with aloe vera gel prevented the development of PCOS in the rats. Aloe vera gel formulation has a protective effect against PCOS by restoring ovarian steroid status and altering steroidogenic activity, which can be attributed to plant compounds such as phytosterols and phytophenols in aloe vera gel. Aloe vera gel acts directly on key enzymes such as 3β HSD, reducing enzyme activity and modulating estradiol formation (Maharjan *et al*., 2010). In another study evaluating the effect of aloe vera hydroalcoholic extract in the treatment of PCOS in rats, the animals were divided into five groups: control group, PCOS group (daily intake of 4 mg/kg estradiol valerate intramuscularly), and treatment groups 1, 2, and 3, each given respective daily doses of 100, 200 and 400 mg/kg of yellow aloe extract intraperitoneally, in addition to 4 mg/kg of estradiol valerate. Estrogen concentration in the PCOS group increased significantly compared to the control group and decreased significantly in treatment groups 2 and 3 compared to controls. The concentration of progesterone in the PCOS group and all treatment groups had a significant decrease compared to the control group. Thus, aloe vera seems to have positive effects on fertility and improvement of polycystic ovary syndrome (Hemayatkhah-Jahromi & Rahmanian-Koushkaki, 2016).

### Chamomile

Chamomile, with the scientific name of *Chamomilla matricaria*, is a perennial plant of the Asteraceae family. Chamomile extract contains flavonoid compounds and antioxidants such as gallic acid, kamazelin, farnesene, matricin, coumarin derivatives, apigenin, and choline, which have special anti-inflammatory effects (Srivastava *et al*., 2009). The antispasmodic effects of chamomile reduce menstrual cramps and the risk of preterm labor in women. Chamomile is used to treat skin complications and eczema due to its stimulating effect on leukocytes (macrophages and B lymphocytes) (McKay & Blumberg, 2006; Farideh *et al*., 2010).

To determine the effect of chamomile on PCOS, 30 adult Wistar rats were studied. The animals were divided into two groups: The first group was the control group and PCP was induced in the second group by injecting estradiol valerate. The PCOS rats were treated with intraperitoneal injections of chamomile alcoholic extract for ten days at different doses (25, 50, and 75 mg/kg). The results of tissue and hormonal tests showed that chamomile could reduce the PCOS symptoms in ovarian tissue and increase the number of uterine follicles, as well as improve LH secretion (Farideh *et al*., 2010).

### Chaste tree

Chaste tree, with the scientific name *Vitex agnus-castus* L, belongs to the Verbenaceae family. The most important chemical compounds in the woody limbs, leaves, and fruits of the *Vitex agnus-castus* are iridoids, flavonoids, alkaloids, glycosides, and steroids. Flavonoids are the main constituents of different parts of this plant (Russo & Galletti, 1996). *Vitex agnus-castus* extract has anti-inflammatory and wound healing effects (Sarma *et al*., 1990). The essential oil of its leaves has antimicrobial activity on gram-positive and negative bacteria (Russo & Galletti, 1996). The extract of this plant corrects high estrogen-dependent hormonal imbalance or premenstrual syndrome and reduces the associated symptoms (Reynolds, 1996). The extract of the *Vitex agnus-castus* changes the amount of sex hormones and their balance. The plant extract also affects the balance of hormones, changing the estrone-to-progesterone ratio, making progesterone from estrogen (Russo & Galletti, 1996). *Vitex agnus-castus* extract can improve the status of these hormones in polycystic animals by altering the secretion of sex hormones.

A total of 93 women who were planning to become pregnant in the next 6-36 months were divided into two groups: a control group and a group that underwent a dietary treatment with *Vitex agnus-castus* extract, vitamins, and minerals. After 3 months, progesterone levels increased in the early luteal phase in the women and menstrual periods returned to normal. Fourteen of the 53 women who received the supplementary diet became pregnant, while 4 of the 40 women in the control group became pregnant. The recommended dose was 500-1000 mg of dried plant (Westphal *et al*., 2006). In a study on the effect of hydroalcoholic extract of *Vitex agnus-castus* fruit on changes in sex hormones on PCOS in Sprague Dawley rats, the animals were divided into four groups: a control group, a treatment group (receiving plant extract at a dose of 365 kg/mg for 30 days), a PCOS group (to induce PCOS using 1 kg/mg letrozole for 28 days), and a PCT group (treated orally with plant extract for 30 days after the PCOS induction). The results showed that after administration of letrozole, estrogen and progesterone levels decreased in the PCOS group, but testosterone and DHEA levels increased. *Vitex agnus-castus* extract in the treatment control group did not cause significant changes in the evaluated hormones. Administration of the extract of this plant increased the serum level of progesterone and decreased testosterone in the animals with PCOS, but did not affect the estradiol and DHEA levels. The plant extract may also increase the activity of the aromatase enzyme and reduce testosterone levels by aromatizing testosterone and converting it to estradiol.

### Cinnamon

Cinnamon, also called *Cinnamomum zeylanicum*, belongs to the Lauraceae family. This plant is one of the oldest and most important herbal medicines used in traditional medicine. Different parts of this plant, including its skin, have many healing properties. The medicinal value of this plant is mostly due to its volatile oil. The main constituents of this essential oil, including cinnamaldehyde, eugenol, and safrole, have insulin-like activity (Singh *et al*., 2007). Cinnamon extract increases glucose uptake and glycogen production and increases insulin receptor phosphorylation, increasing insulin sensitivity (Uslu *et al*., 2018). Cinnamon reduces blood fat and glucose levels (Sheng *et al*., 2008) and prevents the oxidation of organic matter in the body and the reduction of free radicals due to its strong antioxidant properties (Saei Ghare Naz & Ramezani Tehrani, 2021). In addition, in lowering blood sugar and blood lipids, cinnamon can be useful for regulating the menstrual cycle and improving gynecological problems, and breathing and digestive disorders (Heshmati *et al*., 2021).

In one study, 15 women with PCOS took 333 mg of cinnamon extract as oral capsules three times a day for 8 weeks. Insulin sensitivity tests were performed on the patients before and after the administration of cinnamon extract. The results showed a significant reduction in insulin in the patients 2 hours after ingestion of cinnamon extract. Cinnamon decreased insulin in patients by increasing phosphatidylinvestyl 4-kinase activity (Wang *et al*., 2007).

### Ginseng

Ginseng, with the scientific name of *Panax ginseng*, is a fragrant and durable medicinal plant belonging to the genus Panax and the Araliaceae family. This plant has high antioxidant properties and increases the body’s resistance (Helms, 2004). The antioxidant properties of ginseng are induced by stimulating the activity of antioxidant enzymes such as glutathione and superoxide dismutase. Due to its antioxidant activity, this plant can eliminate superoxide and also prevents lipid peroxidation in cell membranes by inhibiting the activity of hydroxyl radicals and anions (Attele *et al*., 1999). This formulation significantly reduces plasma LH levels and is thus effective in improving endocrine status in the treatment of ovulation disorders in patients with PCOS (Andhalkar *et al*., 2021).

A study examined the effect of ginseng on Sprague Dawley rats with PCOS induced by intramuscular injection of estradiol. The animals were divided into three groups: a control group (estradiol injection), an estradiol injection and ginseng treatment group, and an oil treatment group. Nerve growth factor (NGF) and ovarian morphology were studied in the animals. The results showed that in animals with PCOS, NGF increased in the ovaries and brain, and ginseng decreased NGF (Pak *et al*., 2005). In another study, 50 adult rats were divided into a control group, a PCOS group (letrozole treatment), and 3 treatment groups receiving metformin (500 mg/kg), ginseng extract (500 mg/kg), and metformin with ginseng. Administrations were done by gavage for 28 days. Letrozole alone was found to cause a significant increase in serum levels of testosterone, LH, number of cystic follicles, and body weight, and a significant decrease in the amount of estrogen, progesterone, and ovarian follicles compared to the control group. However, in letrozole-treated rats, metformin and ginseng alone and together prevented the effect of letrozole on the serum levels of estrogen and progesterone, testosterone, LH, ovarian and cystic follicles. The difference between the effect of metformin and ginseng was not significant (Shabani & Hosseini, 2017).

### Stachys lavandulifolia

Stachys, with the scientific name of *Stachys lavandulifolia*, belongs to the Lamiaceae family. This plant induces menstruation, is abortive, reduces the duration and severity of primary dysmenorrhea pain, and is a rheumatic analgesic. Chemical analysis of Stachys lavandulifolia plant shows that this plant contains flavonoids, ethanoids, trenapenoids, saponins, quinine, iridoids, phenolic acids, and diterpenoids. Other compounds of the plant are Myrsen (20%), Agnol (18%), Alpha Pyogenin (13.2%), and Gamma Morolen (7%) (Kolivand *et al*., 2017). Flavonoid apigenin, one of the compounds in this plant, is an estrogen present in aromatic plants. Although this compound is less active than its isoflavonoid homologues, its estrogenic properties have been proven. The estrogenic compounds in *Stachys lavandulifolia* extract occupy estrogen receptors and reduce estrogen function (Pahlevani *et al*., 2016).

In a 2013 study, 66 patients with PCOS were randomly divided into two groups. The patients in the intervention group (n=33) were treated with *Stachys lavandulifolia* brewed tea three times a day (5 g each time) for three months. The patients in the control group received medroxyprogesterone acetate (MPA) at a dose of 10 mg per night for 10 nights a month for three months. There was no significant difference in the prevalence of abnormal uterine bleeding (AUB) in both groups. The prevalence of side effects was lower in the intervention group compared to the control group. However, this difference was not significant. Therefore, it was concluded that *Stachys lavandulifolia* could be used as an alternative to MPA in the management of PCOS-induced AUB (Jalilian *et al*., 2013).

The effect of *Stachys lavandulifolia* extract on endometrial histological parameters of the PCOS model of Sprague Dawley rats was investigated. PCOS was induced by estradiol valerate at a dose of 4 mg via intramuscular injections in the animals. The animals were divided into 6 groups: a control group without drug, a PCOS group receiving the solvent drug, a PCOS group receiving *Stachys lavandulifolia* extract (225, 450, and 900 mg/kg), and PCOS groups receiving clomiphene citrate (1.5 mg/kg). The extract was injected intraperitoneally to the tested rats for 4 estrus cycles (16 days). The rats in the PCOS group showed an increase in the height of endometrial epithelial cells and glandular epithelial cells and a decrease in endometrial thickness and inner diameter of the glands, although these changes were not significant. However, parameter number of glands in the PCOS group showed a significant decrease. In addition, hyperplastic changes were seen in 40% of rats in the PCOS group. Treatment with *Stachys lavandulifolia* with different concentrations and clomiphene citrate brought the variables of endometrial epithelial cell height, endometrial thickness, glandular epithelial cell height, endothelial diameter, and the number of glands closer to the control group, but these changes were not significant. Treatment with a concentration of 900 kg/kg of *Stachys lavandulifolia* and clomiphene reduced hyperplasia in 35% and 33.3% of the animals, respectively. Therefore, *Stachys lavandulifolia* extract is effective in changing endometrial tissue parameters in PCOS (Pahlevani *et al*., 2016).

### Fennel

Fennel, with the scientific name of *Foeniculum vulgare*, is an herbaceous plant from the Apiaceae family. Fennel is known as an estrogenic compound and is used in traditional medicine to treat many digestive, endocrine, reproductive, and menstrual disorders. Fennel and its compounds have antibacterial, antifungal, antioxidant, antithrombotic, anti-diabetic, and anti-tumor activity (Ghavi *et al*., 2019). The most important and abundant compound in fennel is a substance called Trans anethole, which has been introduced as an estrogenic active agent. Other aromatic compounds in fennel such as dianethole, photoanethole, estrogole, fenchine, and p-anisaldehyde act as estrogenic biologically active molecules (Parandin & Yousofvand, 2018).

The effect of metformin and fennel on uterine tissue and serum concentrations of estrogen and progesterone was investigated in rats with PCOS. A total of 40 female rats were divided into 5 groups: (1) a control group (receiving normal water and food), (2) a PCOS group (induction of PCOS by intramuscular injection of estradiol valrite at 4 mg/kg body weight), (3) a PCOS group treated with fennel at a dose of 150 mg/kg body weight after induction of polycystic ovary syndrome, (4) a PCOS group treated with fennel at a dose of 100 mg/kg body weight after PCOS induction, and (5) a PCOS group treated with metformin at a dose of 111 mg/kg body weight. After a 63-day treatment period, blood samples were taken from all rats for biochemical analysis, and their uterine tissue was removed for histological studies. The results showed that fennel decreased estrogen, uterine epithelial thickness, and increased progesterone and uterine endometrial thickness in PCOS rats. Therefore, fennel can have a protective effect on the uterine tissue of rats with PCOS (Meena *et al*., 2019; Sadr Fozalaee *et al*., 2015).

### Licorice

Licorice, with the scientific name of *Glycyrrhiza glabra* L, belongs to the Fabaceae family, which is used in traditional medicine for wound healing, pain relief, cough, and gastritis relief. This plant is important because it contains important medicinal compounds in its roots, including flavonoids, sterols, gums, starches, and essential oils. Sterols or phytoestrogens can lower triglycerides and cholesterol. The main active ingredient in licorice root is glycerin, which is 50 times sweeter than sucrose. Research has shown that glycerin has a mineralocorticoid-like effect by inhibiting the 11beta-hydroxysteroid dehydrogenase type 2 (11betaHSD2) enzyme. By inhibiting the metabolism of glucocorticoids, their levels in blood increase. Furthermore, glucocorticoids stimulate insulin secretion and thus lower blood sugar (Andhalkar *et al*., 2021; Barazesh *et al*., 2016; El-Saber Batiha *et al*., 2020).

The effects of administration of licorice root hydroalcoholic extract on blood sugar, triglyceride, and cholesterol levels were investigated in 50 Sprague rats with polycystic ovary syndrome. The studied groups were: a normal group receiving letrozole normal saline (2 ml/kg) orally daily for 21 days, a letrozole group that received letrozole (1 mg/kg) orally for 21 days followed by normal saline (2 ml/kg) orally daily for 30 days, and treatment groups 1 and 2 receiving letrozole (1 mg/kg) orally for 21 days, and then licorice root hydroalcoholic extract (200 and 400 mg/kg), respectively, daily for 30 days. The results showed that the mean serum glucose level in the letrozole group increased compared to the normal group (*p*<0.01) and decreased in treatment groups 1 and 2 compared to the letrozole group (*p*>0.05). The mean serum triglyceride and cholesterol levels did not show a significant difference in the studied groups. Therefore, licorice root extract improves the adverse effects of diabetes due to polycystic ovary syndrome (Barazesh *et al*., 2016).

### Flax

Flax, with the scientific name *Limum usitatissimum*, belongs to the Linaceae family (Zohary *et al*., 2012). Flaxseed is rich in fat, protein, and fiber in the diet. Flaxseed contains on average 30-40% fat, 20-25% protein, 20-28% fiber, 4-8% moisture, and 3-4% ash, vitamins A, B, D, E, minerals, and amino acid (Oomah & Mazza, 1997). Flaxseed oil is rich in linolenic acid, omega-3 fatty acids, lignans, and mucilage (Foster *et al*., 2009). The concentration of α-linolenic acid (omega-3 unsaturated fatty acid) as an anti-cancer agent in flaxseed oil is about 40-60% (Williams *et al*., 2007). Flaxseed has antifungal, antibacterial, and antiviral properties (Kitts *et al*., 1999) and is a rich source of lignans with antioxidant and hypolipidemic effects (Newairy & Abdou, 2009). Flaxseed contains about 27 types of cancer inhibitors including SDG and Matairsinol (Jenab & Thompson, 1996).

Flax seed is rich in lignans, and studies have shown that it lowers androgen levels in men with prostate cancer. Androgen in the body is increased due to polycystic ovaries and is associated with hirsutism, menstrual disorders, and obesity. A study examined the effect of flaxseed taken as an oral supplement 30 grams per day on hormone levels in a 31-year-old woman with PCOS. Over 4 months, the patient took a supplement containing 83% of the flaxseed dose. Height, weight, and fasting blood samples were assessed at the beginning and after 4 months of follow-up. The results were reported pairwise: BMI (36 m/kg^2^ *vs*. 35.7 m/kg^2^), insulin (5.1 µIU/ml *vs*. 7 µIU/ml), total serum testosterone (150 ng/dl *vs*. 45 ng/dl), free serum testosterone (4.7 ng/dl *vs*. 0.5 ng/dl), and percentage of free testosterone (3.1% *vs*. 1.1%). The results showed a significant decrease in body mass index, insulin, serum testosterone, and free serum testosterone. The patient also reported a reduction in hirsutism at the end of the period, and androgen levels decreased with decreasing hirsutism (Emam *et al*., 2021; Nowak *et al*., 2007).

### N-acetylcysteine

N-acetylcysteine is an amino acid derived from cysteine and is the acetylated form of both the amino acid L-cysteine and the reduced form of glutathione. It can stimulate insulin secretion from pancreatic cells as well as regulate insulin receptors at the erythrocyte level. It is also a powerful antioxidant compound and a potential agent in the treatment of cancer and other diseases associated with oxidative stress. The main activity of N-acetylcysteine is due to the sulfhydryl group that can increase the activity of the glutathione-S-transfer enzyme and protect the cell and its membrane against oxidative agents. It can also cleanse and detoxify cells and reduce serum homocysteine levels. N-acetylcysteine can be used as a supplement to increase fertility, improve ovulation, and improve lipid profile and insulin resistance in women with PCOS (Behrouzi Lak *et al*., 2017; Shirani *et al*., 2019).

A clinical trial included 108 women with PCOS randomly divided into two groups, taking metformin or N-acetylcysteine. Eight weeks after the start of the study, the variables between the two groups showed no significant change, but after 12 weeks, the effect of N-acetylcysteine was significantly higher in hirsutism and blood sugar levels than metformin. Besides, after the first and second cycles, the rate of ovulation and pregnancy was higher in the women in the N-acetylcysteine receptor group than in the women in the metformin receptor group. Overall, in comparison with metformin, administration of N-acetylcysteine as a supplement to clomiphene citrate can improve the hormonal profile and hyperinsulinemia and regulate homocysteine (Nemati *et al*., 2017).

### D-chiro-inositol

D-chiro-inositol (DCI) is produced from the decomposition of phytic acids in fruits, legumes, nuts, and grains (Watson, 2011). Inositols are involved in a variety of functions, including cell membrane formation, cell growth and survival (morphogenesis, rearrangement of cytoskeleton, and regulation of cell proliferation), intracellular signaling, peripheral nerve development and function, ossification, and reproduction (Januszewski *et al.,* 2019). D-chiro-inositol can improve regular menstruation and insulin resistance in PCOS patients (Nordio & Proietti, 2012).

In one study, 44 obese women with PCOS were selected and their serum and glucose steroids tested before and after oral administration of 1,200 mg D-chiro-inositol per day for 6 to 8 weeks, and serum progesterone concentrations were evaluated weekly. Ovulation was also measured. The results showed that serum-free testosterone, plasma triglyceride levels, and blood pressure decreased in 19 of the 22 women who received D-chiro-inositol (Nestler *et al*., 1999). In another study, women with PCOS who had insulin resistance were treated with myoinositol and D-chiro-inositol, and combination therapy with both substances. The results showed that after three months, combination therapy with myoinositol and D-chiro-inositol was more effective in reducing PCOS and the risk of metabolic syndrome in overweight women (Nordio & Proietti, 2012). Significant reduction in body weight, FSH, LH, and plasma insulin concentration was observed after 3 and 6 months following treatment with D-chiro-inositol (Januszewski *et al*., 2019).

## CONCLUSION

Polycystic ovary syndrome (PCOS) is an endocrine disorder that leads to female infertility. Various studies have shown that herbal medicines with minimal side effects play an important role in the treatment of PCOS, although more time is required to treat PCOS with herbal medicines. The effect of herbs in the treatment of PCOS can be attributed to the strengthening of the immune system and the regulation of the menstrual cycle without changes in hormonal levels. Table 1 shows the specifications of the medicinal plants reviewed in this study.

**Table 1:** The specifications of the medicinal plants

Plant	Scientific name	Family	Effective compound(s)	The impact on PCOS
Aloe vera	*Aloe arborescen*	Liliaceae	Polysaccharide compounds	Beneficial and supportive effects on ovarian tissue and folliculogenesis
Chamomile	*Chamomilla matricaria*	Asteraceae	Flavonoids and antioxidants such as gallic acid, kamazelin, farnesene, matricin, coumarin derivatives, apigenin, and choline	Reducing PCOS symptoms in ovarian tissue and increasing the number of uterine follicles, as well as helping with LH secretion.
Chaste tree	*Vitex agnus* - *castus* L	Verbenaceae	Flavonoids, aromatase enzymes	Improving the status of sex hormones in polycystic animals by altering the secretion of these hormones (reducing testosterone)
Cinnamon	*Cinnamomum zeylanicum*	Lauraceae	Phosphatidylinvestyl 4-kinase	Increasing glucose uptake, glycogen production and, insulin receptor phosphorylation, improving insulin sensitivity, and reducing lipid and blood glucose levels
Ginseng	*Panax ginseng*	Araliaceae	Glutathione and superoxide dismutase	Affecting corticotropin and cortisol, regulating the immune response, producing antioxidants, having neuroendocrine activity, regulating carbohydrate and fat metabolism
Stachys	*Stachys lavandulifolia*	Lamiaceae	Flavonoid epigenin	By occupying estrogen receptors, it reduces estrogen function (reduction of abnormal uterine bleeding)
Fennel	*Foeniculum vulgare*	Apiaceae	Trans anethole-, photoanethole, estragole, fenchine, and P-anisaldehyde Dianethole	Decreasing estrogen and uterine lining thickness, increasing progesterone and uterine endometrial thickness
Liquorice	*Glycyrrhiza glabra* L	Fabaceae	Glycyrrhiza	Improving the adverse effects of PCOS-induced diabetes
Flax	*Limum usitatissimum*	Linaceae	Lignan	Decreasing androgen and hirsutism
N-acetylcysteine	N-acetylcysteine	Amino acids	Sulfhydryl	Increased chance of fertility, improved ovulation, improved lipid profile, insulin resistance in women with PCOS
D-chiro-inositol	D-chiro-inositol	Inositols	D-chiro-inositol	Decreasing insulin resistance

## References

[r1] Akbar S (2020). Handbook of 200 Medicinal Plants.

[r2] Amini L, Tehranian N, Movahedin M, Ramezani Tehrani F (2015). Effect of Calligonum Comosum on Ovarian Histology of Polycystic
Ovary mouse model. J Med Plants.

[r3] Andhalkar S, Chaware V, Redasani V (2021). A Review on Medicinal Plants of Natural Origin for Treatment of
Polycystic Ovarian Syndrome (PCOS). Asian J Pharm Res Dev.

[r4] Attele AS, Wu JA, Yuan CS (1999). Ginseng pharmacology: multiple constituents and multiple
actions. Biochem Pharmacol.

[r5] Barazesh F, Mirzaei A, Abbasian Z, Ghavami Zadeh M (2016). The Effect of Hydroalcoholic Extract of Glycyrrhizaglabra L.
(liquorice) Root on Serum Level of Glucose, Triglyceride, and Cholesterol in
Polycystic Ovary Syndrome Induced by Letrozole in Rats. Armaghan Danesh.

[r6] Behrouzi Lak T, Hajshafiha M, Nanbakhsh F, Oshnouei S (2017). N-acetyl cysteine in ovulation induction of PCOS women underwent
intrauterine insemination: An RCT. Int J Reprod Biomed.

[r7] El-Saber Batiha G, Magdy Beshbishy A, El-Mleeh A, Abdel-Daim MM, Prasad Devkota H (2020). Traditional Uses, Bioactive Chemical Constituents, and
Pharmacological and Toxicological Activities of Glycyrrhiza glabra
L. Biomolecules.

[r8] Emam SR, Abd-Elsalam RM, Azouz AA, Ali SE, El Badawy SA, Ibrahim MA, Hassan BB, Issa MY, Elmosalamy SH (2021). Linum usitatissimum seeds oil down-regulates mRNA expression for
the steroidogenic acute regulatory protein and Cyp11A1 genes, ameliorating
letrezole-induced polycystic ovarian syndrome in a rat model. J Physiol Pharmacol.

[r9] Farideh ZZ, Bagher M, Ashraf A, Akram A, Kazem M (2010). Effects of chamomile extract on biochemical and clinical
parameters in a rat model of polycystic ovary syndrome. J Reprod Infertil.

[r10] Foster R, Williamson CS, Lunn J (2009). Culinary oils and their health effects. Nutr Bull.

[r11] Ghavi F, Taghizadeh M, Taebi M, Abdolahian S (2019). Effect of Foeniculum vulgare essence on symptoms of polycystic
ovarian syndrome (PCOS): A randomized double-blind, placebo-controlled
trial. J Herbal Med.

[r12] Helms S (2004). Cancer prevention and therapeutics: Panax ginseng. Altern Med Rev.

[r13] Hemayatkhah-Jahromi V, Rahmanian-Koushkaki M (2016). Effect of hydro-alcoholic extract of Aloe vera L. on polycystic
ovary syndrome in rat. Feyz.

[r14] Heshmati J, Sepidarkish M, Morvaridzadeh M, Farsi F, Tripathi N, Razavi M, Rezaeinejad M (2021). The effect of cinnamon supplementation on glycemic control in
women with polycystic ovary syndrome: A systematic review and
meta-analysis. J Food Biochem.

[r15] Jalilian N, Modarresi M, Rezaie M, Ghaderi L, Bozorgmanesh M (2013). Phytotherapeutic management of polycystic ovary syndrome: role of
aerial parts of wood betony (Stachys lavandulifolia). Phytother Res.

[r16] Januszewski M, Issat T, Jakimiuk AA, Santor-Zaczynska M, Jakimiuk AJ (2019). Metabolic and hormonal effects of a combined Myo-inositol and
d-chiro-inositol therapy on patients with polycystic ovary syndrome
(PCOS). Ginekol Pol.

[r17] Jenab M, Thompson LU (1996). The influence of flaxseed and lignans on colon carcinogenesis and
beta-glucuronidase activity. Carcinogenesis.

[r18] Kitts DD, Yuan YV, Wijewickreme AN, Thompson LU (1999). Antioxidant activity of the flaxseed lignan secoisolariciresinol
diglycoside and its mammalian lignan metabolites enterodiol and
enterolactone. Mol Cell Biochem.

[r19] Kolivand M, Keramat A, Khosravi A (2017). The Effect of Herbal Teas on Management of Polycystic Ovary
Syndrome: A Systematic Review. J Midwifery Reprod Health.

[r20] Maharjan R, Nagar PS, Nampoothiri L (2010). Effect of Aloe barbadensis Mill. formulation on Letrozole induced
polycystic ovarian syndrome rat model. J Ayurveda Integr Med.

[r21] McKay DL, Blumberg JB (2006). A review of the bioactivity and potential health benefits of
peppermint tea (Mentha piperita L.). Phytother Res.

[r22] Meena M, Sharma M, Dhakar A, Singh C, Sharma L, Purvia RP (2019). Role of foeniculum vulgare in PCOS - A review
article. World J Pharm Res.

[r23] Mezzetti M, Minuti A, Bionaz M, Piccioli-Cappelli F, Trevisi E (2020). Effects of Aloe arborescens Whole Plant Homogenate on Lipid
Metabolism, Inflammatory Conditions and Liver Function of Dairy Cows during
the Transition Period. Animals (Basel).

[r24] Nasiri Amiri F, Ramezani Tehrani F, Simbar M, Mohammadpour Thamtan RA (2013). Concerns of Women with Polycystic Ovary Syndrome: A Qualitative
Study. Iran J Endocrinol Metab.

[r25] Nemati M, Nemati S, Taheri AM, Heidari B (2017). Comparison of metformin and N-acetyl cysteine, as an adjuvant to
clomiphene citrate, in clomiphene-resistant women with polycystic ovary
syndrome. J Gynecol Obstet Hum Reprod.

[r26] Nestler JE, Jakubowicz DJ, Reamer P, Gunn RD, Allan G (1999). Ovulatory and metabolic effects of D-chiro-inositol in the
polycystic ovary syndrome. N Engl J Med.

[r27] Newairy AS, Abdou HM (2009). Protective role of flax lignans against lead acetate induced
oxidative damage and hyperlipidemia in rats. Food Chem Toxicol.

[r28] Nordio M, Proietti E (2012). The combined therapy with myo-inositol and D-chiro-inositol
reduces the risk of metabolic disease in PCOS overweight patients compared
to myo-inositol supplementation alone. Eur Rev Med Pharmacol Sci.

[r29] Nowak DA, Snyder DC, Brown AJ, Demark-Wahnefried W (2007). The Effect of Flaxseed Supplementation on Hormonal Levels
Associated with Polycystic Ovarian Syndrome: A Case Study. Curr Top Nutraceutical Res.

[r30] Oomah BD, Mazza G (1997). Effect of Dehulling on Chemical Composition and Physical
Properties of Flaxseed. LWT Food Sci Technol.

[r31] Pahlevani P, Mosavi S, Rastgoo Haghi AR, Lahotian H, Esna Ashari F, Alizadeh Z (2016). Study of the Effects of Stachys Lvandulifolia Alcoholic Extract
on Histomorphometry of Endometrium in Polycystic Ovarian Syndrome Rat
Model. Avicenna J Clin Med.

[r32] Pak SC, Lim SC, Nah SY, Lee J, Hill JA, Bae CS (2005). Role of Korean red ginseng total saponins in rat infertility
induced by polycystic ovaries. Fertil Steril.

[r33] Parandin R, Yousofvand N (2018). In Utero and Lactational Effects of Aqueous Foeniculum Vulgare
(Fennel) Seed Extract on Puberty Timing, Estrus Cycle and Sexual Behavior in
Mice. J Arak Uni Med Sci.

[r34] Rashidi S, Farajee H, Jahanbin D, Mirfardi A (2012). Evaluation of Knowledge, Belief, and Operation of Yasouj People
Towards Pharmaceutical Plants. J Med Plants.

[r35] Reynolds JEF (1996). Martindale: The Extra Pharmacopoeia.

[r36] Russo M, Galletti GC (1996). Medicinal properties and chemical composition of Vitex
agnus-castus L.: A review. Acta Hortic.

[r37] Sadr Fozalaee S, Farokhi F, Khaneshi F (2015). The Effect of Metformin and Aqueous Extract Foeniculumvulgare
(Fennel) on Endometrial Histomorphometry and the Level of Steroid Hormones
in Rats with Polycystic Ovary Syndrome. Qom Univ Med Sci J.

[r38] Saei Ghare Naz M, Ramezani Tehrani F (2021). Effect of cinnamon on polycystic ovary syndrome (PCOS): A
Systematic Review. Iran J Obstet Gynecol Fertil.

[r39] Sarma SP, Aithal KS, Srinivasan KK, Udupa AL, Kumar V, Kulkarni D, Rajagopal PK (1990). Anti-inflammatory and wound healing activities of the crude
alcoholic extract and flavonoids of Vitex leucoxylon. Fitoterapia.

[r40] Shabani T, Hosseini S (2017). Comparison of the Hydro-Alcoholic Extract of Ginseng Root with
Metformin in Rats with Polycystic Ovary Syndrome. Armaghan Danesh.

[r41] Sheng X, Zhang Y, Gong Z, Huang C, Zang YQ (2008). Improved Insulin Resistance and Lipid Metabolism by Cinnamon
Extract through Activation of Peroxisome Proliferator-Activated
Receptors. PPAR Res.

[r42] Shirani M, Nouri M, Askari G (2019). Effect of N-acetylcysteine supplementation on Polycystic Ovary
Syndrome (PCOS) in infertile women: A systematic review of clinical
trials. Clin Excell.

[r43] Singh G, Maurya S, DeLampasona MP, Catalan CA (2007). A comparison of chemical, antioxidant and antimicrobial studies
of cinnamon leaf and bark volatile oils, oleoresins and their
constituents. Food Chem Toxicol.

[r44] Srivastava JK, Pandey M, Gupta S (2009). Chamomile, a novel and selective COX-2 inhibitor with
anti-inflammatory activity. Life Sci.

[r45] Uslu GA, Gelen V, Uslu H, Özen H (2018). Effects of Cinnamomum cassia extract on oxidative stress,
immunoreactivity of iNOS and impaired thoracic aortic reactivity induced by
type II diabetes in rats. Braz J Pharm Sci.

[r46] Wang JG, Anderson RA, Graham GM 3rd, Chu MC, Sauer MV, Guarnaccia MM, Lobo RA (2007). The effect of cinnamon extract on insulin resistance parameters
in polycystic ovary syndrome: a pilot study. Fertil Steril.

[r47] Watson RR (2011). Complementary and Alternative Therapies and the Aging Population: An
Evidence-based Approach.

[r48] Westphal LM, Polan ML, Trant AS (2006). Double-blind, placebo-controlled study of Fertilityblend: a
nutritional supplement for improving fertility in women. Clin Exp Obstet Gynecol.

[r49] Williams D, Verghese M, Walker LT, Boateng J, Shackelford L, Chawan CB (2007). Flax seed oil and flax seed meal reduce the formation of aberrant
crypt foci (ACF) in azoxymethane-induced colon cancer in Fisher 344 male
rats. Food Chem Toxicol.

[r50] Yagi A (2015). Putative Prophylaxes of Aloe vera for Age-Related
Diseases. J Gastroenterol Hepatol Res.

[r51] Zohary D, Hopf M, Weiss E (2012). Domestication of Plants in the Old World: The origin and spread of
domesticated plants in Southwest Asia, Europe, and the Mediterranean
Basin.

